# Multidisciplinary Management of Acute Esophageal Necrosis Secondary to Alcoholic Lactic Acidosis: A Case Report

**DOI:** 10.3390/reports8010025

**Published:** 2025-02-19

**Authors:** Luigi Orsini, Alberto Martino, Ornella Picascia, Marco Di Serafino, Giovanni Lombardi

**Affiliations:** 1 Department of Gastroenterology and Digestive Endoscopy, AORN “Antonio Cardarelli”, 80131 Napoli, Italy; luigi.orsini@aocardarelli.it (L.O.); giovanni.lombardi@aocardarelli.it (G.L.); 2 Department of General and Emergency Radiology, AORN “Antonio Cardarelli”, 80131 Napoli, Italy; ornella.picascia@aocardarelli.it (O.P.); marco.diserafino@aocardarelli.it (M.D.S.)

**Keywords:** upper gastrointestinal bleeding, non variceal upper gastrointestinal bleeding, black esophagus, acute esophageal necrosis, alcoholic lactic acidosis

## Abstract

**Background and Clinical Significance**: Acute esophageal necrosis (AEN), or black esophagus, is an extraordinary rare source of acute upper gastrointestinal bleeding. Its pathogenesis is still poorly understood, whereas etiology seems to be multifactorial, mainly involving esophageal ischemia, increased acid reflux, and reduced mucosal defenses. Although alcohol abuse has been reported to be a common trigger factor, only one case of AEN due to severe alcoholic lactic acidosis has been described up to date. **Case Presentation**: Herein, we describe a case of a non-cirrhotic 61-year-old lady with a history of chronic alcohol abuse, who was admitted to the Emergency Room due to upper gastrointestinal (GI) bleeding. AEN caused by severe alcoholic lactic acidosis was promptly diagnosed by subsequent investigations, including blood test, urinalysis, computed tomography, and upper GI endoscopy. The treatment involved a multidisciplinary, aggressive medical approach, which included one hemodialysis session. **Conclusions**: This is the second documented case of AEN secondary to alcoholic lactic acidosis, successfully treated with a previously unreported aggressive multidisciplinary approach, involving one hemodialysis session. It highlights the value of a multidisciplinary approach in managing such complex and rare conditions.

## 1. Introduction and Clinical Significance

First reported in 1990 by Goldenberg and colleagues [1], acute esophageal necrosis (AEN), also defined as black esophagus, is a rare and potentially life-threatening condition, characterized endoscopically by black-colored esophageal mucosa abruptly ending at the gastro-esophageal junction, and histologically by extensive mucosal necrosis [2,3,4]. Upper gastrointestinal bleeding is the most common clinical presentation of AEN [2,5,6].

The exact pathogenesis is still unknown, but its etiology seems to be multifactorial, encompassing esophageal ischemia, increased acid reflux, and reduced mucosal defenses [2,3,4]. Various conditions have been associated with the development of AEN, such as hemodynamic compromise, some medications, diabetic ketoacidosis, inflammatory states, malignancy, surgery, gastric outlet obstruction, and chronic alcohol abuse [2,3,4]. Although the latter has been shown to be the predisposing factor in up to 25% of patients with AEN [7,8], until now only one case of AEN secondary to alcoholic lactic acidosis has been reported [9].

To date, there is no specific treatment for AEN. Hydration, acid suppression, bowel rest, antibiotics in case of infection, and proper treatment of the underlying illness currently represent the mainstay of AEN management [4,10,11].

However, given the rarity of the disease, optimal management is still unclear.

The reported mortality of AEN is high, ranging from 30% to 50%, mainly reflecting the severity of the underlying illness [5,11,12].

Herein, we describe a case of AEN caused by severe alcoholic lactic acidosis, successfully treated by means of a previously unreported multidisciplinary, aggressive medical approach, involving one hemodialysis session.

## 2. Case Presentation

A 61-year-old lady with a history of chronic alcohol abuse was admitted to the Emergency Room due to a 24 h severe epigastric pain associated with episodes of vomiting and recent onset of active coffee-ground emesis with signs of hemodynamic instability. Her past medical history included alcoholic liver disease in the absence of cirrhosis. She declared alcohol binging during the previous days. Conversely, the ingestion of any caustic agents, methanol, and drugs was denied.

### 2.1. Diagnostic Evaluation

At the time of the admission, she was pale, afebrile, eupneic in room air, with a regular pulse of 110 bpm, and a blood pressure of 100/70 mmHg. Mild conscious impairment was present. Her physical examination was negative for any evidence of portal hypertension or other stigmata of chronic liver disease. Abdominal examination revealed a distended abdomen and a moderate epigastric tenderness. The rectal examination did not exhibit melena or blood. A Foley catheter was inserted into the bladder, and normochromic urine was returned. A naso-gastric tube was placed and about 500 mL of coffee-ground material with blood traces and clots was removed.

Arterial blood gas measurements showed the following: pH 7.22, PaCO_2_ 16.2 mmHg, PaO_2_ 107.7 mmHg, AG 26.3 mmol/L, HCO_3_^−^ 6.7 mmol/L, lactate > 20.0 mmol/L, glucose 62 mg/dL.

Laboratory examinations were remarkable for moderate anemia (hemoglobin 8.5 g/dL) and mild hepatic cytolysis (AST 68 UI/L, ALT 70 UI/L). All remaining laboratory examinations, including liver and renal function tests, were within normal values. Blood tests for ethanol, isopropanol, methanol, and ethylene glycol screening were all negative. Urinalysis revealed neither ketones nor oxalate crystals. Urine drug screen was negative.

**Figure 1:**
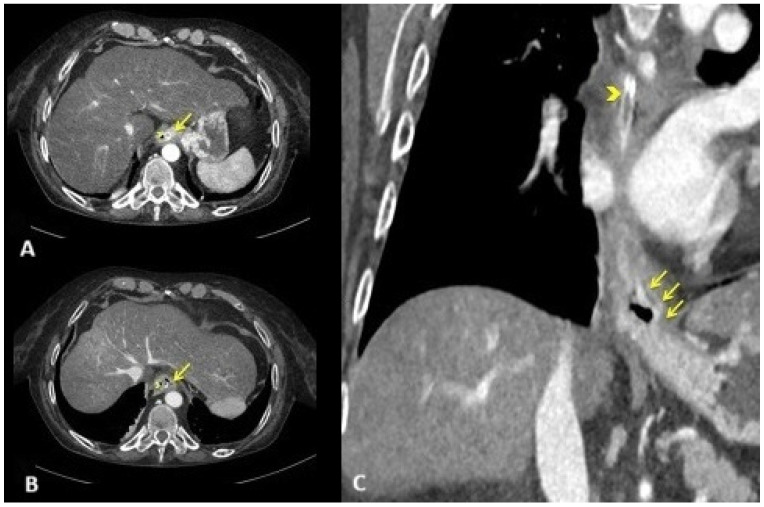
Emergent computed tomography angiography. Axial arterial (**A**) CT scan at the level of the gastro-esophageal junction and venous (**B**) CT scan at the level of lower thoracic esophagus showing circumferential thickening of the esophageal wall. Note the mucosal enhancement ((**A**); yellow arrow) with wall hypoattenuation, producing the target sign ((**B**); yellow arrow). Coronal venous CT reconstruction showing extension of esophagitis predominantly to the distal third of the esophagus, including the gastro-esophageal junction ((**C**); yellow arrows). Nasogastric tube ((**A**–**C**); yellow arrowhead).

Fluids with 80 mL of 7% sodium bicarbonate, thiamine, and high-dose pantoprazole were administered intravenously. Then, an emergent computed tomography angiography was performed, demonstrating a grossly dilated gastric cavity alongside a thickened distal esophagus with evidence of transmural inflammation, in the absence of free air or active bleeding (Figure 1).

**Figure 2:**
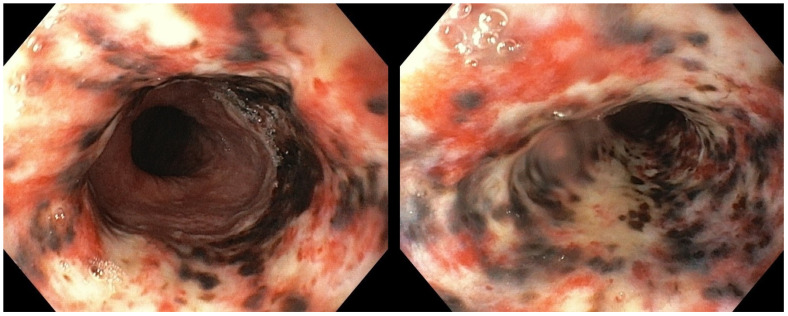
Urgent esophagogastroduodenoscopy showing diffusely black-colored mucosa at the level of the middle and the distal esophagus, abruptly ending at the gastro-esophageal junction.

The patient was admitted to our Bleeding Unit. After the hemodynamic stabilization, an urgent esophagogastroduodenoscopy (EGD) under monitored anesthesia care was carried out. The endoscopic examination showed diffuse, circumferential, black-colored, friable mucosa at the middle and the distal esophagus, in the absence of signs of active bleeding. Gastric mucosa was spared (Figure 2). Esophageal biopsies were not taken at this time due to concern about a possible perforation.

### 2.2. Treatment and Outcome

Treatment with total parenteral nutrition and a high dose of pantoprazole continuous infusion was started. However, despite aggressive intravenous hydration, repeated arterial blood gas analysis at 2, 3, and 5 h after the patient’s admission, showed the persistence of severe lactic acidosis (pH 7.28, anion gap 26.0 mmol/L, lactate 19.9 mmol/L, HCO_3_^−^ 8.1 mmol/L). Thus, following a multidisciplinary evaluation which involved the nephrologist and the emergency physician, an urgent dialysis was planned. A single 3 h and 30 min hemodialysis session was carried out, resulting in nearly normalization of the severe lactic acidosis (pH 7.46, anion gap 11 mmol/L, lactates 7.4 mmol/L, HCO_3_^−^ 23.5 mmol/L).

**Figure 3:**
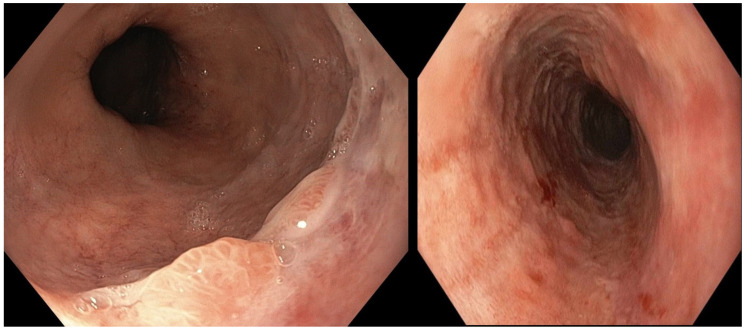
Follow-up esophagogastroduodenoscopy showing a nearly complete resolution of the esophageal necrosis, with mild hyperemia and congestion at the level of the middle and the distal esophagus.

Post-operative stay was uneventful. Following the normal intestinal canalization and a clinical and laboratory improvement, on day 5, oral diet was gradually resumed. On day 7, EGD was repeated, showing nearly complete healing of the AEN, with only mild hyperemia and congestion at the medial and the distal esophagus, in the absence of strictures or other complications (Figure 3). Esophageal biopsies were taken, evidencing mild epithelial non-specific inflammation only. The patient was discharged home on day 8, and no complications were observed up to the 6-month follow-up.

## 3. Discussion

AEN is an extremely rare disease, with a reported incidence ranging from 0.0125% to 0.2% [12,13,14], which mainly presents with upper gastrointestinal bleeding [2,5,6]. It is associated with poor prognosis. Indeed, the overall mortality is approximately 30–50% [5,11,12], mainly related to underlying critical illness. Conversely, the mortality directly related to AEN is relatively low, at about 6% [11]. The main complications of AEN include esophageal perforation and stenosis. The first one is the most severe and has a reported incidence of approximately 5–7%, whereas the last one is a late complication encountered in about 10% of patients on follow-up endoscopy [15]. Although the exact pathogenesis of AEN is still unknown, its etiology seems to be multifactorial, mainly involving esophageal ischemia, increased acid reflux, and reduced mucosal defenses [4]. Several conditions have been reported to be associated with the development of AEN, including alcohol abuse, which has been identified as the triggering factor in up to 25% of patients [7,8]. However, to date, only one case of AEN secondary to alcoholic lactic acidosis has been reported [9].

Conservative supportive care, primarily including hydration, acid suppression, bowel rest, and antibiotics in case of infection, along with an adequate treatment of the underlying medical conditions, is currently suggested in almost all cases [4,10,11]. However, given the rarity of the disease, the optimal treatment strategy is still unknown.

As previously reported by Endo and colleagues [8], the leading pathogenesis of our case might be accounted for by the systemic hypoperfusion induced by severe alcoholic lactic acidosis. However, in our case, an early response of severe alcoholic lactic acidosis to vigorous intravenous hydration was not observed. Lactic acidosis is associated with sudden death in alcoholics and has profound effects on the circulation system and organ perfusion [16,17,18]. Thus, following a multidisciplinary evaluation, a single hemodialysis session was performed, aimed at improving the patient’s prognosis [19,20]. A prompt resolution of the lactic acidosis was subsequently observed. Moreover, the resolution of EAN was observed early at one week, and neither early nor late complications were encountered.

To the best of our knowledge, the presented case is the second report of AEN secondary to severe alcoholic lactic acidosis, which was effectively treated with a previously unreported aggressive multidisciplinary approach, involving one hemodialysis session.

## 4. Conclusions

Early recognition and aggressive multidisciplinary approach are crucial for the successful management of AEN secondary to severe alcoholic lactic acidosis and therefore to improving outcomes of this life-threatening disease.

## Data Availability

The data presented in this study are available on request from the corresponding author due to privacy concerns.
